# Family Risk for Depression and Prioritization of Religion or Spirituality: Early Neurophysiological Modulations of Motivated Attention

**DOI:** 10.3389/fnhum.2019.00436

**Published:** 2019-12-17

**Authors:** Jürgen Kayser, Craig E. Tenke, Connie Svob, Marc J. Gameroff, Lisa Miller, Jamie Skipper, Virginia Warner, Priya Wickramaratne, Myrna M. Weissman

**Affiliations:** ^1^Division of Cognitive Neuroscience, New York State Psychiatric Institute, New York, NY, United States; ^2^Department of Psychiatry, Columbia University Vagelos College of Physicians and Surgeons, New York, NY, United States; ^3^Division of Translational Epidemiology, New York State Psychiatric Institute, New York, NY, United States; ^4^Spirituality Mind Body Institute, Teachers College, Columbia University, New York, NY, United States; ^5^Mailman School of Public Health, Columbia University, New York, NY, United States

**Keywords:** depression risk, emotional lateralization, event-related potential (ERP), religion/spirituality, source localization, surface Laplacian, visual half-field paradigm

## Abstract

The personal importance of religion or spirituality (R/S) has been associated with a lower risk for major depression (MDD), suicidal behavior, reduced cortical thinning and increased posterior EEG alpha, which has also been linked to antidepressant treatment response in MDD. Building on prior event-related potential (ERP) findings using an emotional hemifield paradigm, this study examined whether abnormal early (preconscious) responsivity to negative arousing stimuli, which is indicative of right parietotemporal dysfunction in both MDD patients and individuals at clinical high risk for MDD, is likewise moderated by R/S. We reanalyzed 72-channel ERP data from 127 individuals at high or low family risk for MDD (Kayser et al., 2017, *NeuroImage Clin.* 14, 692–707) after R/S stratification (low R/S importance, low/high risk, *n* = 38/61; high R/S importance, *n* = 15/13). ERPs were transformed to reference-free current source density (CSD) and quantified by temporal principal components analysis (tPCA). This report focused on N2 sink (peak latency 212 ms), the earliest prominent CSD-tPCA component previously found to be sensitive to emotional content. While overall N2 sink reflected activation of occipitotemporal cortex (prestriate/cuneus), as estimated via a distributed inverse solution, affective significance was marked by a relative (i.e., superimposed) positivity. Statistical analyses employed both non-parametric permutation tests and repeated measures ANOVA for mixed factorial designs with unstructured covariance matrix, including sex, age, and clinical covariates. Participants with low R/S importance, independent of risk status, showed greater ERP responsivity to negative than neutral stimuli, particularly over the right hemisphere. In contrast, early emotional ERP responsivity and asymmetry was substantially reduced for high risk individuals with high R/S importance, however, enhanced for low risk individuals with high R/S importance. Hemifield modulations of these effects (i.e., emotional ERP enhancements with left visual field/right hemisphere stimulus presentations) further corroborated these observations. Results suggest down-regulation of a right-lateralized network for salience detection at an early processing stage in high risk and high R/S importance individuals, presumably to prevent overactivation of ventral brain regions further downstream. These findings may point to a neurophysiological mechanism underlying resilience of families at risk for depression with high R/S prioritization.

## Introduction

Religion (or religiousness) and spirituality (R/S) are widely considered core constituents of all human societies (e.g., Inzlicht et al., 2009, 2011; Crescentini et al., 2015). They can provide for many individuals purpose to human existence, as well as resources to cope with stressful life experiences which appear to have a beneficial impact on mental (e.g., depression) and physical (e.g., cardiovascular) health (Koenig et al., 2001, 2012; Hill and Pargament, 2003; Cobb et al., 2012). Despite the omnipresence of R/S and its putative health relevance, and a growing interest of relating R/S to brain mechanisms on a conceptual level (Fingelkurts and Fingelkurts, 2009; van Elk and Aleman, 2017), empirical neuroscientific research addressing these relations and the underlying neurobiological processes has been scarce.

Over the last 20 years, our group has focused on the protective mechanism afforded by the personal importance of R/S during an ongoing longitudinal study of families at high and low risk for depression (e.g., Weissman et al., 1997, 2005, 2006, 2016). Maternal R/S importance and mother-offspring concordance of R/S was associated with a 90% reduction of risk for incurring a depressive episode at 10-year follow-up (Miller et al., 1997), and offspring with high R/S importance had a 75% risk reduction of experiencing MDD 10 years later, particularly for those who were at high family risk because of a depressed parent (Miller et al., 2012). Parental R/S importance was associated with lower risk for suicidal behavior in offspring (Svob et al., 2018). High R/S importance was also associated with less cortical thinning (Miller et al., 2014), a putative morphologic endophenotype of familial risk for MDD (Peterson et al., 2009). Further implicating protective benefits of R/S importance for persons at high risk for depression, those who self-reported high R/S importance, when compared those who did not, had larger pial surface representing the outer boundary area between gray matter and cerebrospinal fluid (Liu et al., 2017). Moreover, posterior EEG alpha oscillations at rest, a putative biomarker of antidepressant treatment response (Tenke et al., 2011), was greater in individuals who rated R/S as highly important compared to those who did not, provided ratings were obtained during early stages of ontogenetic development (Tenke et al., 2013, 2017). Likewise, *increased* resting-state functional MRI default mode network connectivity in high compared to low risk individuals (Posner et al., 2016) was reported to be relatively *decreased* (i.e., lowered) with greater R/S importance (Svob et al., 2016).

Whereas the above cited reports investigated rather persistent, time-invariant neurobiological markers (i.e., structural MRI and posterior alpha; see Hao et al., 2017; Tenke et al., 2018), an even more critical question is how *transient* brain responses to *salient* stimuli are affected by R/S importance, as these processes reflect ongoing (state) mechanisms of coping with events in daily life. Dysfunctions in emotion processing and regulation are considered to be a core deficit of mood disorders (e.g., Gross and Munoz, 1995; Rive et al., 2013), and mindfulness meditation, which may be closely linked to R/S importance, has been claimed to exert beneficial effects on physical and mental health (Chiesa and Serretti, 2010; Hölzel et al., 2011; Lomas et al., 2015; Lutz et al., 2015; Tang et al., 2015). Over several decades, affective neuroscience has identified key modules for emotional processing and self-awareness, including amygdala, striatum, nucleus accumbens, anterior insula, orbitofrontal, ventromedial, prefrontal, anterior cingulate, and posterior cingulate cortex (e.g., Davidson et al., 2000; Phan et al., 2002; Phillips et al., 2003a, b; Pessoa, 2008; Price and Drevets, 2012).

The right occipitoparietotemporal cortex, specifically the rTPJ (e.g., Liotti and Tucker, 1995; Tucker, 2015), plays a critical role in the early detection of affective stimulus significance, a critical mechanism for survival guiding approach and withdrawal behavior (e.g., Lang et al., 1998; Bradley, 2009). These right hemisphere regions are embedded within a network involving cortical (anterior insula, anterior cingulate cortex) and subcortical (amygdala, striatum) structures for detecting emotional and reward saliency (Corbetta and Shulman, 2002; Lutz et al., 2015). Using rTMS, Crescentini et al. (2014, 2015) reported a direct link between R/S self-representations and neuronal activation of the right IPL, where increasing IPL excitability yielded a decrease of implicit R/S. Using fMRI, Johnson et al. (2014) found reduced BOLD activity in primary visual cortex in response to religious symbols with negative valence, and this activity was correlated with a measure of R/S importance, suggesting that a person’s R/S beliefs interact with salience detection and processing at an early stage in the processing hierarchy of the visual system. Interestingly, after reviewing the literature on beneficial clinical effects of mindfulness practices, Chiesa et al. (2013) considered the possibility that mindfulness meditation practices are, depending on the practitioner’s skill level, differentially linked to ‘top–down’ (short-term meditation practice) versus ‘bottom–up’ (long-term) control processes of emotion regulation (e.g., Ochsner and Gross, 2005; Ochsner et al., 2009).

Electrophysiological measures, such as ERPs, are particularly suited to study transient brain responses to motivationally salient stimuli because they (1) *directly* reflect neuronal activation and (2) allow characterization of *consecutive* processing stages with millisecond temporal resolution. However, few studies have examined ERP effects of religiosity or R/S importance. Inzlicht et al. (2009) found that stronger religious zeal and greater belief in God was associated with reduced ERN, considered a correlate of performance monitoring and self-regulation originating from anterior cingulate cortex (e.g., Gehring and Knight, 2000). The authors interpreted the reduced ERN as neurophysiological evidence for a protective buffer against anxiety (i.e., minimizing error experience) caused by a cognitive style associated with strong convictions. A follow-up study (Good et al., 2015) implied that decreased affective responses to errors are linked to focusing on God’s love and forgiveness rather than on God’s wrath and punishment. In another study, Thiruchselvam et al. (2017) reported that increased late ERP positivity to faces was less affected by peer ratings of attractiveness in non-religious as compared to religious undergraduates, suggesting higher social conformity in the latter group.

Given the overall scarcity of reports examining the effects of religiosity or R/S importance using ERP measures, the current study sought to build on a broad literature relying on automatic (bottom–up) ERP responses to affective stimuli (e.g., for reviews, see Olofsson et al., 2008; Hajcak et al., 2012). The most consistent finding is what is known as the LPP, an increased posterior positivity to emotional (pleasant or unpleasant) than neutral pictures, which emerges around 200 ms after stimulus onset and closely covaries with arousal (e.g., Kayser et al., 1997; Cuthbert et al., 2000; Schupp et al., 2000; Keil et al., 2008). It is assumed that the increase in positivity reflects an extra allocation of attentional resources to stimuli that intrinsically engage motivational brain circuits via re-entrant projections to and from brain structures outside the visual system, such as the lateral prefrontal cortex and amygdala (e.g., Vuilleumier and Driver, 2007; Pessoa, 2008; Bradley, 2009; Keil et al., 2009; Pourtois et al., 2013). Conversely, multiple prefrontal regions may exert inhibitory influences, allowing down-regulation of emotional processing (e.g., Tang et al., 2015). For example, active suppression of emotional arousal in response to unpleasant pictures was accompanied by reduced LPP amplitudes (Moser et al., 2006). Two ERP studies reported that experienced meditators, when compared to control participants without meditation experience, showed attenuated responses to negative pictures, either for the sustained LPP (500–1500 ms time interval, linked-mastoids EEG reference, frontal sites only; Sobolewski et al., 2011) or during an early LPP interval (140–400 ms, average reference; Reva et al., 2014). The latter study also found that the emotional ERP reductions were more prominent over the right than left hemisphere, suggesting that early, right-lateralized ‘bottom–up’ processes of salience detection are modulated by long-term meditators.

Several ERP studies have previously reported evidence of right-lateralized brain activation during an early (preconscious) stage of affective processing (e.g., Kayser et al., 1997, 2000; Junghöfer et al., 2001; Keil et al., 2001, 2002). To specifically probe hemispheric asymmetries of emotional processing in an ongoing study of families at high and low risk for depression (Kayser et al., 2016, 2017), we have employed the visual half-field technique, which exploits the functional neuroanatomy of the visual system. Brief, lateralized stimulus presentations to one hemifield are exclusively projected to the contralateral visual cortex (e.g., left hemifield to right hemisphere), providing direct (immediate) access to the presented information, whereas the ipsilateral hemisphere has only indirect (secondary) access to this information after commissural transfer (e.g., Young, 1982; Springer and Deutsch, 1989). Pairs of pictures depicting facial areas of patients with skin diseases *before* (negative valence) and *after* (neutral) surgical treatment were used as stimulus material during this hemifield paradigm (Kayser et al., 1997, 2000, 2016, 2017). While most studies have manipulated emotional content by using stimuli of the *International Affective Picture System* (IAPS; e.g., Lang et al., 2005; Bradley and Lang, 2007), the advantage and main purpose of this paired stimulus set is isolating the emotional content construct (negative valence, high arousal) from other stimulus characteristics (e.g., content, complexity, luminance, contrast, color), as changes in these properties will also affect early and late ERP components (e.g., Delplanque et al., 2007; Wiens et al., 2011). Self-report ratings of valence and arousal (Bradley and Lang, 1994) for these stimuli indicated a near optimal characterization of the evaluative space for affective stimuli along a negativity dimension (see Figure 1 in Kayser et al., 2016). In support of our hypotheses, we found right-lateralized emotional effects (i.e., a relative positivity for negative than neutral stimuli) for an early ERP component, termed an N2 sink peaking at about 200 ms, which could be attributed to neuronal generator sources within right occipitotemporal cortex using a distributed inverse solution (Kayser et al., 2016). These early emotional ERP asymmetries were further modulated by hemifield, with larger amplitude and asymmetry of emotional effects for left hemifield (right hemisphere) presentations. A follow-up report (Kayser et al., 2017) showed that these early emotional ERP asymmetries were markedly reduced in individuals at high than low family risk for depression, which is in agreement with evidence showing reduced electrophysiological responsivity to affective signals over right temporoparietal regions in MDD patients (e.g., Kayser et al., 2000; Moratti et al., 2008, 2015; Domschke et al., 2015) and individuals at increased risk for MDD (e.g., Kujawa et al., 2015; Nelson et al., 2015, 2016; Weinberg et al., 2015; Gibb et al., 2016); however, hemifield modulations did not differ between risk groups, suggesting top–down rather than bottom-up effects of risk.

If personal importance of R/S is related to or represents a protective mechanism against familial risk for depression (Miller et al., 1997, 2012), one possible means to accomplish a beneficial effect on well-being and mental health would be to modulate or down-regulate (negative) arousal linked to salience detection at an early stage of processing, that is, even before the information reaches conscious awareness (e.g., LeDoux, 2015), as implicated by the ERP findings of Reva et al. (2014). Accordingly, we would predict that activity within the right-lateralized network for salience detection (Corbetta and Shulman, 2002) is disrupted or inhibited in high risk individuals with high importance of R/S, thereby exhibiting reduced early emotional ERP asymmetries and/or reduced hemifield modulations of these effects. The purpose of the present exploratory study was to test these predictions by taking advantage of an existing and already processed 72-channel ERP data set obtained during our emotional hemifield paradigm in a large sample (*N* = 127) of high and low risk individuals (Kayser et al., 2017), who had also provided ratings of R/S importance. Following our prior reports (Miller et al., 1997, 2012, 2014; Tenke et al., 2013, 2017), we stratified the sample into participants who had rated their personal R/S importance as high versus those who did not. This dichotomous R/S variable was then added to the split-plot repeated measures design for N2 sink, that is, the earliest ERP component that had yielded prominent emotional content effects in this hemifield paradigm (Kayser et al., 2016).

## Materials and Methods

### Participants

The sample has been described in detail in our prior report (Kayser et al., 2017). Briefly, it consisted of 127 Caucasian individuals (58 male) between 13 and 59 years of age (*Mean* ± *SD* = 33.2 ± 13.9) who were enrolled in a multi-generation, 35-year longitudinal study of families at high and low risk for major depression (Talati et al., 2013; Weissman et al., 2016). Probands were initially selected for the presence or absence of a lifetime history of MDD from outpatient psychiatric clinics and their urban community in New Haven, CT, United States (Weissman et al., 1982, 1992). The current participants were biological descendants (i.e., the second and third generation) of the original probands (i.e., the first generation). Participants with at least one depressed parent or grandparent were considered at high family risk for MDD (*n* = 74, 32 male, age: 35.1 ± 14.2 years), whereas all others were considered at low family risk for MDD (*n* = 53, 26 male, age: 30.4 ± 13.0). Most participants (*n* = 114, 89.8%) were right-handed (Oldfield, 1971; for further details, see Kayser et al., 2017). Participants had to be older than 12 years and without a history of seizures, head trauma or psychosis to be eligible for the emotional hemifield task.

A comprehensive summary of all relevant clinical assessments has been given in our previous report (Kayser et al., 2017). Briefly, clinicaln assessments, beginning as early as age 6, were obtained from probands, their spouses, offspring and grandchildren at six longitudinal waves using semi-structured interviews. Diagnoses were based on age-appropriate versions of the *Schedule for Affective Disorders and Schizophrenia-Lifetime Version* (Kaufman et al., 1997) using a best estimate procedure (Leckman et al., 1982), allowing lifetime diagnoses of MDD and anxiety disorder (AD). Current depressive and anxiety symptoms were also assessed on all but eight participants using the Hamilton Rating Scales for Depression (Hamilton, 1967) and Anxiety (Hamilton, 1959) for adults and the Children’s Depression Rating Scale (Poznanski et al., 1985) and Revised Child Manifest Anxiety Scale (Perrin and Last, 1992) for children 17 years and under. These data were converted to standard scores (CurrDep_z_, CurrAnx_z_) and the missing data were imputed from the existing data of current depressive and anxiety symptoms (Kayser et al., 2017).

All participants had normal or corrected-to-normal visual acuity. EEG testing was performed at the Psychophysiology Laboratory at New York State Psychiatric Institute (NYSPI) during the sixth wave of assessments. All procedures were approved by the Institutional Review Boards at Yale University and at NYSPI/Columbia University. All participants gave written informed consent (≥18 years) or provided written assent (<18 years; written informed consent from parents) in accordance with the ethical standards specified in the 1964 Declaration of Helsinki.

### Importance of Religion or Spirituality

While most of the original families (Generation 1) were Roman Catholic, the self-identified denomination of the 127 participants (Generations 2 and 3) at the time of data collection was more diverse, including Catholic (*n* = 66, 52.0%), Protestant (*n* = 17, 13.4%), Jewish (*n* = 5, 3.9%), personal beliefs not affiliated with any institutional religion (*n* = 15, 11.8%), Agnostic or Atheist (*n* = 13, 10.2%), and other or refused to answer (*n* = 11, 8.7%).

Data collection in this ongoing longitudinal study (now in Year 40) has typically been separated by approximately 5–10 years increments since Year 10 (i.e., at Years 0, 2, 10, 20, 25, 30, 35, and 40). Beginning with Year 10, participants also rated their personal importance of religion or spirituality (R/S) on a four-level Likert scale, with response options to the question *“How important is religion or spirituality to you?”* ranging from *“not important at all”* to *“highly important.”* This item has been found to show robust correlations with the Fetzer Institute full-scale measure of personal spirituality (see Miller et al., 2014). The terms “religion” and “spirituality” were both included in this question because they are frequently linked together in studies on health (e.g., Koenig et al., 2001). To estimate test-retest reliability of R/S importance, an intraclass correlation coefficient (ICC) was calculated from ratings obtained since Year 10, which were each available for *n* = 62 participants (24% missing data across all waves), yielding an ICC of 0.828, which is considered excellent (Cicchetti, 1994) or good (Koo and Li, 2016).

In accordance with prior reports (Miller et al., 1997, 2012, 2014; Tenke et al., 2013, 2017), the sample was stratified by each participant’s response to this item as either “highly important” (R/S+) or any other response (R/S−). Given our recent findings suggesting that an early stage in the ontogenesis of R/S is critical for its link to posterior resting alpha (Tenke et al., 2017), both R/S importance and EEG alpha being putative markers of resilience against MDD (Tenke et al., 2013), the earliest available importance rating was used for this classification (mean age at R/S importance rating: 19.4 ± 9.9 years). Table 1 shows the resulting stratification of risk status by R/S importance along with core demographics variables.

**TABLE 1:** Crosstabulation of religion/spirituality importance and family risk of MDD with sex and generation, and corresponding means (±SD) of core demographic and clinical variables.

**Importance^a^**			**Sex**		**Generation^b^**			**Age^c^**	**Age_R/S_^d^**	**EHI^e^**	**HRSD^f^**	**HRSA^g^**
				
			**Male**	**Female**	**2nd**	**3rd**	**Total**					
R/S −	Risk	Low	21	17	15	23	38	31.6 ± 13.4	17.5 ± 10.3	70.3 ± 46.6	1.9 ± 5.3	1.0 ± 2.9
		High	26	35	36	25	61	35.7 ± 14.5	22.0 ± 9.7	74.6 ± 47.9	3.8 ± 5.7	2.4 ± 4.2
	Total		47	52	51	48	99	34.1 ± 14.2	20.2 ± 10.1	72.9 ± 47.2	3.1 ± 5.6	1.9 ± 3.8
R/S +	Risk	Low	5	10	3	12	15	27.3 ± 11.6	14.4 ± 8.3	51.0 ± 56.1	1.2 ± 1.7	1.0 ± 2.1
		High	6	7	5	8	13	32.5 ± 13.1	18.8 ± 8.2	66.8 ± 45.6	5.3 ± 9.9	4.2 ± 8.0
	Total		11	17	8	20	28	29.7 ± 12.4	16.4 ± 8.4	58.3 ± 51.2	3.1 ± 7.0	2.5 ± 5.8
Total			58	69	69	58	127	33.2 ± 13.9	19.4 ± 9.9	69.7 ± 48.3	3.1 ± 5.9	2.0 ± 4.3

*^*a*^Personal rating of religion/spirituality as *highly important* (R/S+) or any other (R/S−). ^*b*^Offspring of original proband in generation 2 (child) or 3 (grandchild). ^*c*^Age at EEG testing. ^*d*^Age at R/S importance rating. ^*e*^Laterality quotient of *Edinburgh Handedness Inventory* (Oldfield, 1971) can vary between -100.0 (completely left-handed) and +100.0 (completely right-handed). ^*f*^*Hamilton Rating Scale for Depression* (Hamilton, 1967) and ^*g*^*Anxiety* (Hamilton, 1959); values reflect back-converted *z*-scores (see text, section Participants).*

While there were no significant differences in sex (Fisher’s Exact Test, *p* = 0.59), age, age at R/S importance rating, or handedness with generation included as a covariate (all *F*_[__1_,_122__]_ < 1.3), the sample included more high than low risk participants from the second generation (Fisher’s Exact Test, *p* = 0.02). High compared to low risk participants had more current symptoms of depression (*F*_[__1_,_122__]_ = 4.85, *p* = 0.03) and anxiety (*F*_[__1_,_122__]_ = 4.30, *p* = 0.04), but there were no significant effects involving R/S importance (all *F*_[__1_,_122__]_ ≤ 2.0, all *p* ≥ 0.16). In the R/S low importance group, there was a significantly greater number of high than low risk participants with a lifetime history of MDD (35 vs. 11) compared to those without (27 vs. 26; Fisher’s Exact Test, *p* = 0.007). However, lifetime history of AD was not significantly different for the groups stratified by family risk and R/S importance (Fisher’s Exact Test, *p* = 0.15).

A total of 41 participants (32%) had a lifetime history of substance use disorder, including alcohol, however, there were no significant subgroup differences (R/S−, low/high risk, *n* = 11/21; R/S+, *n* = 4/5; Fisher’s Exact Test, *p* = 0.70). This was also the case when data were limited to current substance use disorder (i.e., within the last 18 months; R/S−, low/high risk, *n* = 2/7; R/S+, *n* = 1/1; Fisher’s Exact Test, *p* = 0.49) or when considering only substance or alcohol use (i.e., without meeting criteria for dependence; R/S−, low/high risk, *n* = 5/10; R/S+, *n* = 2/0; Fisher’s Exact Test, *p* = 0.15). A total of 23 participants (18%) had a lifetime history of nicotine dependence, and there were also no significant subgroup differences (R/S−, low/high risk, *n* = 3/17; R/S+, *n* = 1/2; Fisher’s Exact Test, *p* = 0.45), including when considering only current nicotine dependence (R/S−, low/high risk, *n* = 2/9; R/S+, *n* = 1/1; Fisher’s Exact Test, *p* = 0.42).

Current use of psychotropic medications (i.e., within the last 3 months, including sedatives, stimulants, antidepressants, anticonvulsants, and lithium, but excluding over the counter medications) was observed for 24 participants (19%), revealing no significant subgroup differences (R/S−, low/high risk, *n* = 4/17; R/S+, *n* = 0/3; Fisher’s Exact Test, *p* = 1.0). Current use of non-prescribed substances (i.e., alcohol and illicit substances, including cannabis, cocaine, heroin, etc.) was reported by 11 participants (9%), also revealing no significant subgroup differences (R/S−, low/high risk, *n* = 2/8; R/S+, *n* = 0/1; Fisher’s Exact Test, *p* = 1.0). Finally, current use of medications to treat physical problems (i.e., including cancer, cardiovascular disease, thyroid disorder, arthritis, autoimmune disorder, skin conditions, liver, kidney, allergies or respiratory disease, among others) was reported for 69 participants (54%), which also did not differ between subgroups (R/S−, low/high risk, *n* = 19/38; R/S+, *n* = 5/7; Fisher’s Exact Test, *p* = 0.74).

There were also no differences between participants at high vs. low risk for depression or high vs. low R/S importance in current medication use to treat physical ailings or in use of non-prescribed substances (all *p* ≥ 0.11). As expected, high risk individuals were more likely than those at low risk to use psychotropic medications (Fisher’s Exact Test, *p* = 0.006), however, there were no differences between R/S subgroups (*p* = 0.28).

### Stimuli and Procedure

All considerations pertaining to stimulus selection, characteristics, ratings, underlying rationale and presentation procedure have been detailed previously (Kayser et al., 1997, 2000, 2016). Briefly, stimuli consisted of 16 closely matched pairs of pictures depicting facial areas of patients with dermatological diseases *before* (negative) and *after* (neutral) surgical treatment. Neutral stimuli differed from their negative counterpart only in the emotionally relevant feature but were almost identical in all other aspects. Stimulus ratings via self-assessment manikin (Bradley and Lang, 1994) indicated that negative stimuli were perceived as moderately unpleasant and arousing whereas neutral stimuli were seen as neither pleasant or unpleasant and not arousing (Kayser et al., 2016). Stimuli were briefly presented for 250 ms on a monitor to the left or right hemifield (NeuroScan Inc., 2003) using a pseudo-randomized sequence (i.e., four blocks of 32 trials) with variable intertrial intervals (8–13 s). Participants attended to the stimulus presentations while maintaining fixation but did not respond manually. Trials with horizontal eye movements (saccades) exceeding 2° from fixation during stimulus exposure were rejected.

### Data Acquisition and Processing

ERP acquisition and processing procedures have been described in detail (Kayser et al., 2016). Briefly, 72-channel EEGs were obtained at 1024 samples/s (BioSemi Inc., 2001), followed by identification and elimination or reduction of typical recording artifacts (i.e., blinks, electrolyte bridges, drifts, movements, muscle, etc.). ERP waveforms were computed for all four conditions (i.e., emotional content [negative, neutral] × hemifield [left, right]), low-pass filtered at 12.5 Hz (-24 dB/octave), and transformed into CSD estimates (μV/cm^2^ units; spline flexibility *m* = 4; smoothing constant λ = 2.5 ^∗^ 10^–5^; Kayser and Tenke, 2006a, b, 2015) using spherical splines (Perrin et al., 1989, 1990). CSDs represent reference-free estimates of radial current flow at scalp (i.e., negative [sink] or positive [source] values represent current flow exiting or toward the scalp, respectively) that avoid several pitfalls of volume-conducted surface potentials while also providing sharper topographies and a more focused component structure (i.e., higher temporal resolution; Tenke and Kayser, 2012; Burle et al., 2015; Carvalhaes and de Barros, 2015; Kayser and Tenke, 2015). CSDs were submitted to temporal PCA (tPCA; Kayser and Tenke, 2003) to obtain data-driven summaries of radial current flow, or reference-free ERP components reflecting neuronal generator patterns at scalp (Kayser and Tenke, 2015). While our prior report identified and analyzed three consecutive CSD-tPCA factors peaking between 200 and 700 ms that were robustly linked to emotional content, the present report focuses on the first of these factors that corresponded to a temporoparietal N2 sink (peak latency 212 ms). The superimposed emotional effects (increased sources [positivity] for negative than neutral stimuli) were strongly lateralized to the right hemisphere, and their distributed inverses (sLORETA; Pascual-Marqui, 2002; Tadel et al., 2011; for computational details using CSD-tPCA factor scores, see Supplementary Appendix and Kayser et al., 2016) revealed maximal activations in right occipitotemporal cortex.

### Statistical Analysis

To assess the combined effects of family risk for depression and R/S importance in the context of the emotional hemifield paradigm, the present report adopted the two-pronged analytical approach employed by Kayser et al. (2017). First, differences in emotional content were evaluated for N2 sink factor scores via non-parametric randomization tests (Maris, 2004; Kayser et al., 2007) to probe the entire topography for each subgroup (i.e., R/S importance × risk). Second, N2 sink factor scores were pooled across three lateral-inferior parietooccipital sites over each hemisphere (PO9/10, PO7/8, P7/8) where emotional content effects were most robust (Kayser et al., 2016). Emotional content, visual field, and group effects were then evaluated with repeated measures analysis of variance (ANOVA) for mixed factorial designs (including between- and within-subjects variables as required), using an unstructured covariance matrix (BMDP-5V; Dixon, 1992) and adding sex, age, lifetime history of MDD and AD, and current severity of symptoms for depression and anxiety as covariates. Unlike conventional *F* statistics, this analytical model is based on maximum likelihood estimates and χ^2^ statistics within a linear regression model, which allows the exploration of interaction sources via linear combinations of regression parameters. We employed a conventional level of significance (*p* < 0.05) and report Cohen’s *w* for effect sizes (small = 0.1, medium = 0.3, large = 0.5; Cohen, 1988).

## Results

### Electrophysiological Data

**FIGURE 1:**
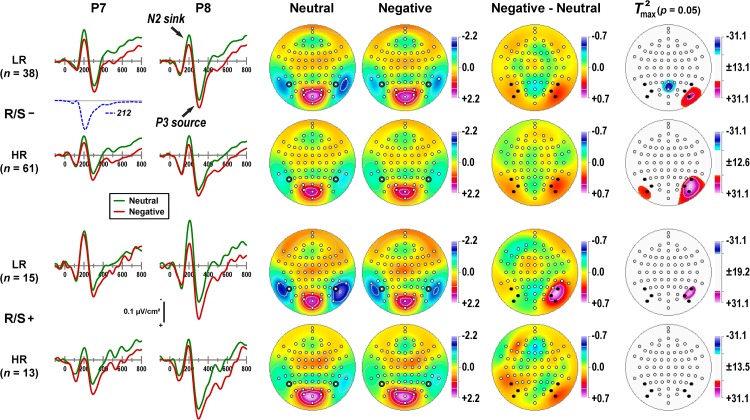
**Columns 1–2:** Current source density (CSD) [μV/cm^2^] waveforms (–100 to 800 ms, 100 ms pre-stimulus baseline) for negative and neutral stimuli (pooled across hemifield) at selected left and right lateral-parietal sites (P7, P8; black circles in columns 3–4) for subgroups stratified by personal importance of religion/spirituality (R/S–: less than highly important; R/S+: highly important) and MDD family risk (LR/HR: low/high risk). Distinct CSD components (N2 sink, P3 source) are labeled in italics at P8 for R/S– individuals at low risk. Factor loadings of the targeted temporal PCA factor corresponding to N2 sink (peak latency 212 ms; Kayser et al., 2016) are shown for comparison (dashed line). **Columns 3–6:** Statistical evaluation of topographic emotional content effects for the corresponding CSD-tPCA factor scores using randomization tests for paired samples (10,000 repetitions) for each subgroup. Shown are the mean factor score topographies for neutral and negative stimuli, the emotional content net effect (negative-minus-neutral), and squared univariate (channel-specific) paired samples *T* statistics thresholded at the 95th quantile (*p* = 0.05) of the corresponding randomization distribution derived from the full sample (maximum of all 72-channel squared univariate paired samples *T* statistics). To facilitate comparisons of the max(*T*^2^) topographies with the underlying difference topographies, the sign of the difference at each site was applied to the respective *T*^2^ value, which is otherwise always positive. Symmetric scales were optimized for score ranges across neutral and negative stimuli and all subgroups. All topographies are two-dimensional representations of spherical spline interpolations (*m* = 2; λ = 0) derived from the mean factors scores or *T*^2^ statistics available for each recording site. Sites marked as black dots (**Columns 5–6**) were used in repeated measures ANOVA.

Figure 1 provides a detailed overview of the emotional content effects related to a distinct N2 sink peaking at about 200 ms, followed by a subsequent P3 source at about 300 ms, as can be seen in the mean CSD waveforms for negative and neutral stimuli at selected lateral parietal sites (P7/8). Furthermore, differences of emotional content emerged around 150 ms, revealing more positive-going CSDs for negative than neutral stimuli that persisted throughout the recording epoch. Notably, this basic CSD component structure and the superimposed emotional effects were present in all subgroups, although to a different degree.

The common variance associated with N2 sink was captured by a CSD-tPCA factor peaking at 212 ms (dashed line in Figure 1; 5.7% total variance; Kayser et al., 2016). The corresponding factor score topographies consisted of prominent lateral temporoparietal sinks that were paired with a mid-parietooccipital P2 source (Figure 1, columns 3-4), implicating a dipolar generator involving secondary visual (prestriate) cortex and cuneus. The topography representing the corresponding emotional content net effect (i.e., negative-minus-neutral) revealed maximum differences at lateral-inferior parietooccipital sites (PO9/10, PO7/8, P7/8) that were larger over the right than left hemisphere (Figure 1, column 5). The non-parametric evaluations of these differences (see permutation tests in Figure 1, column 6) confirmed robust right-greater-than-left asymmetries at this region except for high risk participants with high R/S importance.

**FIGURE 2:**
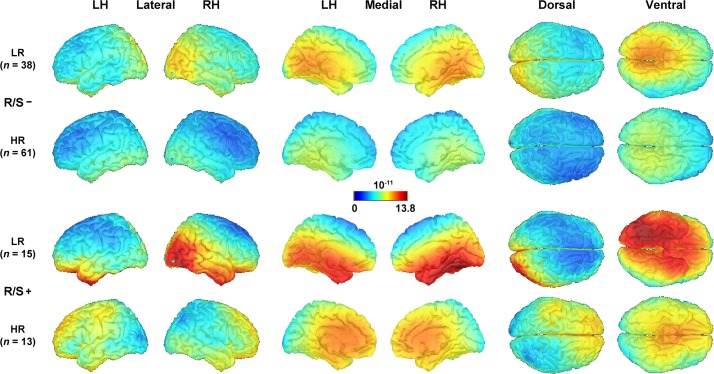
Distributed inverse solutions (sLORETA; Pascual-Marqui, 2002; Tadel et al., 2011) of emotional content net effects (negative-minus-neutral; see Figure 1, column 5) for N2 sink (see Supplementary Appendix for back-projection of CSD-tPCA factors to surface potential data space). An optimized scale range [(pA/m)^1/2^] was used across all subgroups. LH/RH, left/right hemisphere; LR/HR, low/high risk; R/S, religion/spirituality less than highly (–) or highly (+) important.

Further clarification of the putative generators underlying the emotional content net effects in each subgroup was provided by their distributed inverses (sLORETA solutions) shown in Figure 2, which utilize the entire CSD-tPCA-based component topography (for computational details, see Supplementary Appendix and Kayser et al., 2016). While robust asymmetric (i.e., right-greater-than-left) sources in extrastriate cortex can be seen for low risk individuals, these asymmetries were more subtle or absent in high risk individuals (compare rows 1 and 3 vs. rows 2 and 4 in Figure 2). Moreover, these early posterior (i.e., extrastriate cortex) activations were accompanied by strong inferior temporal activations for low risk participants with high R/S importance.

**FIGURE 3:**
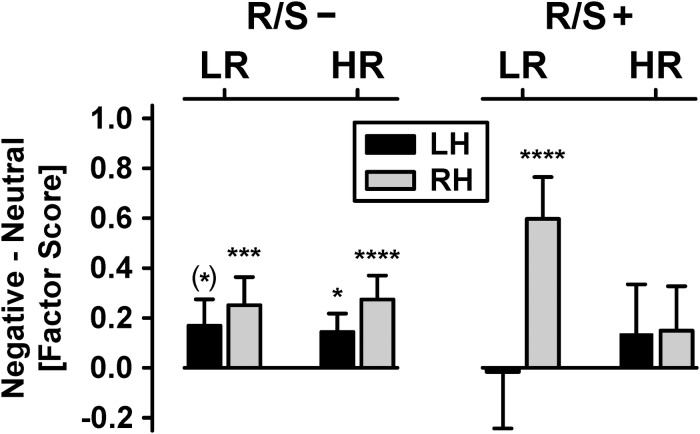
Emotional content net effects (negative-minus-neutral) for each hemisphere (LH/RH: left/right hemisphere), pooled across visual field. Shown are mean (±SEM) differences of N2 sink amplitudes [factor scores] over parietooccipital regions for subgroups stratified by personal importance of religion/spirituality (R/S–: less than highly important; R/S+: highly important) and MDD family risk (LR/HR: low/high risk). Significant simple effects of emotional content (i.e., differences between negative and neutral), corresponding to small to large effects (0.18 ≤ *w* ≤ 0.49), are marked as: (^∗^) *p* < 0.10; ^∗^*p* < 0.05; ^∗∗∗^*p* < 0.001; ^****^p < 0.0001.

Figure 3 shows the mean emotional content net effects at lateral-inferior parietooccipital sites as revealed by the parametric analyses. Most importantly, there was a significant four-way interaction of risk status × R/S importance × emotional content × hemisphere, χ^2^_[__1__]_ = 6.40, *p* = 0.01, *w* = 0.22.^1^ Follow-up analyses revealed a significant emotional content × hemisphere interaction for low risk participants with high R/S importance, χ^2^_[__1__]_ = 16.1, *p* = 0.0001, *w* = 0.36, originating from a robust emotional content effect at the right hemisphere, χ^2^_[__1__]_ = 30.2, *p* < 0.0001, *w* = 0.49, but absence thereof at the left hemisphere. Participants with low R/S importance, and independent of risk status, had significant or marginally significant emotional content effects over both hemispheres, all χ^2^_[__1__]_ ≥ 3.62, all *p* < 0.06, 0.17 ≤ *w* ≤ 0.45, although nevertheless stronger over the right hemisphere, as supported by a marginally significant emotional content × hemisphere interaction for high risk participants with low R/S importance, χ^2^_[__1__]_ = 2.93, *p* = 0.07, *w* = 0.15. In contrast, high risk participants with high R/S importance showed no significant emotional content effects at either hemisphere, both χ^2^_[__1__]_ ≤ 1.64, *p* ≥ 0.20, *w* ≤ 0.11.^2^

**FIGURE 4:**
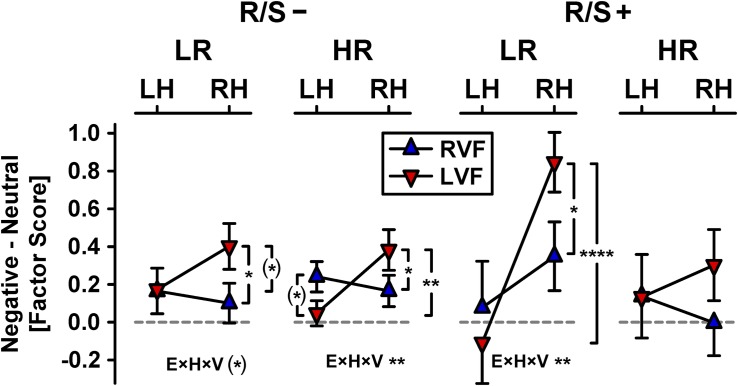
Hemifield modulations of emotional content net effects shown in Figure 3 (RVF/LVF: right/left visual field; all other abbreviations as in Figure 3). Significant pairwise simple effects (indicated by vertical brackets) and emotional content × hemisphere × visual field interactions (E×H×V), the latter corresponding to small to medium effects (0.16 ≤ *w* ≤ 0.28), are marked as: (^∗^)*p* < 0.10; ^∗^*p* < 0.05; ^∗∗^*p* < 0.01; ^****^p < 0.0001.

Figure 4 shows the hemifield modulations of these emotional content × hemisphere effects separately for each subgroup, which revealed enhanced emotional content effects over the right hemisphere for left but not right visual field presentations (Kayser et al., 2016, 2017). Left hemifield presentations showed right-greater-than-left asymmetries of emotional content for participants at low risk with high R/S importance, χ^2^_[__1__]_ = 21.2, *p* < 0.0001, *w* = 0.41 (Figure 4, inverted triangles, column 3), at high risk with low R/S importance, χ^2^_[__1__]_ = 10.6, *p* = 0.001, *w* = 0.29 (column 2), and at low risk with low R/S importance, χ^2^_[__1__]_ = 3.06, *p* = 0.08, *w* = 0.16 (column 1), but not for those at high risk with high R/S, χ^2^_[__1__]_ = 0.58, *p* = 0.45, *w* = 0.07 (column 4). In contrast, right hemifield presentations failed to reveal any significant asymmetric emotional content effects for any subgroup (Figure 4, upright triangles). These group-dependent hemifield modulations of asymmetric emotional content effects were further supported by simple interactions of emotional content, hemisphere, and visual field for individuals at low risk (R/S importance: low, χ^2^_[__1__]_ = 3.17, *p* = 0.08, *w* = 0.16; high, χ^2^_[__1__]_ = 6.87, *p* = 0.009, *w* = 0.23) and high risk with low R/S importance, χ^2^_[__1__]_ = 10.1, *p* = 0.002, *w* = 0.28, but not for those at high risk with high R/S importance, χ^2^_[__1__]_ = 1.22, *p* = 0.27, *w* = 0.1.

## Discussion

The present report evaluated whether individuals at high familial risk for major depression, who rated religion and spirituality as highly important during adolescence and young adulthood, differed in their early, bottom–up processing of unpleasant pictures during an emotional hemifield paradigm (Kayser et al., 2016, 2017). In agreement with prior studies (Miller et al., 2012, 2014; Svob et al., 2016; Liu et al., 2017), R/S importance significantly modulated outcome measures dependent on risk status. Here, overall early emotional ERP responsivity and asymmetry, which was observed for low R/S importance participants independent of risk status, was substantially *diminished* or absent for high risk individuals with high R/S importance; however, it was *enhanced* for low risk individuals with high R/S importance. This is in line with the idea that neuronal activation within the right-lateralized network for salience detection (Corbetta and Shulman, 2002) is down-regulated at an early processing stage in prestriate cortex and cuneus in high risk individuals with high R/S importance. The putative protective function is to prevent or weaken the downstream production of an affective state in response to unpleasant, arousing stimuli involving ventral brain regions (Phillips et al., 2003a, b), which involve inferior temporal and also posterior cingulate cortex for the present emotional hemifield paradigm (Kayser et al., 2016). By contrast, increased allocation of attention to motivationally significant pictures was seen for low risk individuals with high R/S importance, who supposedly would not suffer from experiencing negative arousal; rather, these individuals may enhance processing of motivationally salient stimuli consistent with the idea that mindfulness is associated with a state of relaxed alertness that may promote mental health (e.g., Lomas et al., 2015; Lutz et al., 2015). Interestingly, recent fMRI findings by Miller et al. (2018) implicated reduced activity in left inferior parietal cortex when participants recalled personalized spiritual compared to neutral-relaxing experiences, suggesting prolonged lateralized effects of modulatory mechanisms related to R/S importance.

Our prior report using these data indicated that risk status did not alter hemifield-dependent modulations of early emotional ERP effects (Kayser et al., 2017). The present analysis qualifies this finding because high R/S importance differentially affected risk status, revealing enhanced visual field effects in the low risk group, particularly for left hemifield (right hemisphere) stimulations, whereas hemifield effects were weaker and non-significant in the high risk group (Figure 4). This indicates that R/S importance affects allocation of attentional resources to stimuli that intrinsically engage motivational brain circuits (i.e., motivated attention; Bradley et al., 2003; Vuilleumier and Driver, 2007; Bradley, 2009) at an *early* processing stage, that is, during the stimulus-driven (bottom–up) visual stream that precedes conscious awareness (e.g., LeDoux, 1989, 2015; Tamietto and de Gelder, 2010). Although speculative, this interpretation of our findings is in accordance with the idea that ‘bottom–up’ control processes of emotion regulation are linked to experienced mindfulness meditation (Chiesa et al., 2013), assuming that high R/S importance may reflect a trait aspect or rather stable characteristic of mindfulness. In any case, we note that these conclusions regarding the early onset of these modulatory R/S importance effects are firmly grounded in the temporal sequence of brain activation uniquely afforded by ERPs. This favorable characteristic of ERPs is further improved by their CSD transformations, which provide a more distinct time course than their surface potentials counterparts (Kayser and Tenke, 2006a; Burle et al., 2015), where the signal is impeded (i.e., spatially smeared) by volume conduction (e.g., Nunez and Srinivasan, 2006; Tenke and Kayser, 2012).

Due to the uniqueness of the emotional hemifield paradigm and given the scarceness of prior ERP research with religiosity or the R/S importance construct, it is difficult to integrate the present findings within the broader literature. The association between reduced ERN and firmness of religious belief by Inzlicht et al. (2009) and Good et al. (2015) are consistent with the present results if interpreted as R/S importance affording a protective buffer (i.e., against anxiety or negative affective arousal). In a neuroscience-based account, Inzlicht et al. (2011) suggested that religion is widespread among humans because it fulfills the need for *meaning* (i.e., the perceived coherence between beliefs, salient goals, and perceptions of the environment), making the world appear ordered, controlled, and understandable. Although these researchers have focused on brain activity attributed to anterior cingulate cortex and processes linked to cognitive (self-)control, their “motivated meaning-making” account stresses that religion is a motivated process – this aligns with the idea that differences in personal R/S importance are associated with bottom-up processes of motivated attention (Bradley, 2009), as studied here. Our findings are also in agreement with fMRI findings by Johnson et al. (2014) suggesting that R/S importance affects salience detection at an early stage of the visual system processing hierarchy. Moreover, our findings appear to be in line with rTMS findings by Crescentini et al. (2014, 2015) that suggest a link between the R/S construct and differential activation of right inferior parietal cortex. Clearly, more hypothesis-driven research is needed in the fields of social and affective neuroscience to help deepen our understanding of the relationship between R/S importance, brain activation, and health benefits. However, the present findings allow for the generation of specific testable predictions, namely up- versus down-regulation of early, right-lateralized activity associated with salience detection in individuals with high personal importance of religion or spirituality, depending on their family risk status for depression.

The study has several limitations. First, the assessment of personal R/S importance relies on a single self-report item, which may be an inadequate representation of what comprises religiosity and spirituality (e.g., Hill and Pargament, 2003). However, a more recent wave of this longitudinal study (*N* = 282) employed a comprehensive survey that included several validated scales assessing religious beliefs and experiences and used an exploratory factor analysis to uncover latent R/S constructs that would covary with the R/S importance item (Svob et al., 2019). The first factor (15.8% explained variance) was directly related to the R/S importance item (*r* = 0.82), as well as personal relationship with the Divine, forgiveness by God, religious activities, and religious coping. At the same time, this R/S factor precluded other R/S aspects, including gratitude, altruism, and social support. The R/S factor was reliability reproduced for key subgroups, that is, for generations 2 (*n* = 140) and 3 (*n* = 99), and for high (*n* = 150) and low (*n* = 89) risk individuals, thereby affirming adequate single-item construct validity. The R/S factor observed in this study also closely matched one of five invariant factors identified across three diverse cultures (China, India, United States) by McClintock et al. (2016). That factor, termed Religious and Spiritual Reflection and Commitment, likewise correlated robustly (*r* = 0.79) with McClintock et al.’s (2016) measure of R/S importance, further suggesting that this single self-report item may be adequate for use in health studies lacking the resources for more extensive measures.

Second, due to the cross-sectional design of the current report (i.e., ERP recordings for the emotional hemifield task were only obtained at Year 30; e.g., Talati et al., 2013), it is unclear whether the differential electrophysiological effects observed for high versus low R/S importance are directly linked to specific functional outcomes (e.g., suicidal ideation, cardiovascular health), as such relationships are only implied by familial risk status. Third, the generalizability of the findings is limited by the lack of ethnic, socioeconomic and religious diversity (i.e., the sample was predominantly Caucasian, middle class and Catholic), as all participants were part of a three-generation longitudinal, cohort study that started over 30 years ago (Weissman et al., 1982); still, the sample’s homogeneity also constitutes a strength. Fourth, given that loss of pleasure (anhedonia) is a distinct feature of depression (e.g., Watson et al., 1988), resulting in blunted positive affect and responsivity to reward, the lack of pleasant stimuli may be viewed as a limitation. However, the inclusion of appetitive stimuli will also require the inclusion of corresponding ‘neutral’ stimuli matched for content, as discussed previously (Kayser et al., 2000), which is a significant advantage of the present negative/neutral stimulus pairs.

## Conclusion

Personal importance of religion or spirituality was found to be differentially associated with automatic, preconscious processing of unpleasant stimuli (motivated attention) in individuals at high and low familial risk for depression. For participants indicating high personal importance of R/S in adolescence or young adulthood, emotional ERPs characterizing early (200 ms), right-lateralized emotional arousal of occipitotemporal (extrastriate) cortex (Kayser et al., 2016) were *enhanced* for individuals at low risk but were *reduced* for individuals at high risk. These findings are consistent with prior clinical and neurophysiological evidence suggesting that personal R/S importance may function as a protective buffer against stressful (lifetime) events (i.e., resilience against MDD; e.g., Miller et al., 1997; Svob et al., 2018), presumably by preventing harmful, affective (over-)arousal downstream. Furthermore, the findings implicate a specific neurofunctional mechanism of emotion regulation (Rive et al., 2013; Tang et al., 2015) underlying certain health benefits linked to personal importance of religion and spirituality.

## Data Availability Statement

The datasets for this study will not be made publicly available because data were obtained as part of an ongoing, multi-generational study of families at risk for depression that started in 1982 before there was data sharing; therefore, consent was not obtained. Public data sharing, even anonymously, is restricted by participants’ informed consent. We are in the process of obtaining consent. This will take several years as we enroll subjects into our new study. Interested readers may contact the Principal Investigator, MW, for more information, or by visiting the study’s website at: http://highriskdepression.org.

## Ethics Statement

All procedures of this research were approved by the Institutional Review Boards at Yale University and at New York State Psychiatric Institute/Columbia University. All participants gave written informed consent (≥18 years) or provided written assent (<18 years; written informed consent from parents) in accordance with the ethical standards specified in the 1964 Declaration of Helsinki.

## Author Contributions

JK and CT: design and conceptual idea for reanalysis of existing data. JK: conceptualization and development of emotional hemifield paradigm, data analysis, figures, tables, and supplementary appendix, and drafting and revising the manuscript. MG, JS, and VW: maintenance of clinical database. CS, LM, PW, and MW: conceptualization of single-item measure for assessing personal importance of religion and spirituality. All living authors have critically reviewed the work and provided their final approval for publication.

## Conflict of Interest

For activities unrelated to the current research, the authors report the following financial disclosures during the past 3 years. JK: funding from NIMH and John Templeton Foundation. LM: funding and royalties from American Psychological Association Press, Oxford University Press, Picadore Press, John Templeton Foundation, and Rockefeller Philanthropic Associates. MW: funding from NIMH, the Templeton Foundation and royalties for publications from Perseus Press, Oxford University Press, American Psychiatric Association Press and for the Social Adjustment Scale from Multihealth Systems Inc. None of these pose a conflict of interest. The remaining authors declare that the research was conducted in the absence of any commercial or financial relationships that could be construed as a potential conflict of interest.

## Footnotes

1 To address the issue of multiple comparisons in multiway ANOVA designs (Cramer et al., 2016), the false discovery rate (FDR; Benjamini and Hochberg, 1995) was calculated for the list of *p*-values (all main effects and interactions). Critically, the four-way interaction of risk status × R/S importance × emotional content × hemisphere remained significant after FDR correction (*p* = 0.04).

2 Despite concerns about small cell sizes (Table 1), given the higher prevalence of MDD in women than men (Weissman and Klerman, 1977; Weissman et al., 1984), a repeated measures ANOVA was computed with sex as an additional between-subjects factor. First, the strength of the risk status × R/S importance × emotional content × hemisphere interaction increased substantially, χ^2^_[__1__]_ = 10.25, *p* = 0.001, suggesting that variance associated with sex differences weakened this effect. Second, this analysis yielded three significant effects involving sex: sex × emotional content × hemisphere, χ^2^_[__1__]_ = 4.99, *p* = 0.03; sex × R/S importance × emotional content × hemisphere, χ^2^_[__1__]_ = 4.70, *p* = 0.03; and risk status × R/S importance × sex × hemisphere, χ^2^_[__1__]_ = 10.0, *p* = 0.001. Only the first two interactions, which include emotional content and hemisphere, are relevant to this study. The emotional content × hemisphere interaction, originating from larger emotional content effects (i.e., negative being more positive than neutral) over the right than left hemisphere, was seen for each sex, however, more robust for males than females. R/S importance further qualified this three-way interaction in that these effects were primarily driven by male participants with high R/S importance.

## Abbreviations

CSD: current source density
EEG: electroencephalogram
ERN: error-related negativity
ERP: event-related potentials
IPL: inferior parietal lobe
LPP: late positive potential
MDD: major depressive disorder
PCA: principal components analysis
rTPJ: right temporoparietal junction
R/S: religion/spirituality
rTMS: repetitive transcranial magnetic stimulation
sLORETA: standardized low resolution brain electromagnetic tomography
tPCA: temporal principal components analysis.
