# Supplementary material for: Family Risk for Depression and Prioritization of Religion or Spirituality: Early Neurophysiological Modulations of Motivated Attention
Source: Front Hum Neurosci. 2019 Dec 17;13:436. doi: 10.3389/fnhum.2019.00436 (PMC6927907; doi:10.3389/fnhum.2019.00436)
Supplement: Supplementary file 1 [file Data_Sheet_1.pdf]

Appendix

Below we outline the back-projection of CSD-tPCA (current source density - temporal principal components analysis) factors into surface potential (ERP) data space, which is based on the Matlab code published in the appendix of Kayser and Tenke (2003). The computation of factor scores requires a factor score coefficient matrix, which is directly derived from the factor loadings matrix (see Matlab code lines 25-27 on p. 2323 in Kayser & Tenke, 2003; function *erpPCA.m* is also available at http://psychophysiology.cpmc.columbia.edu/mmedia/Kayser2003a/Appendix.html).

```
1  DataREST = Data;                           % surface potential data matrix
2  LRcsd = LR;                                % Varimax-rotated CSD-PCA factor loadings
3  [cases,vars] = size(DataREST);             % determine matrix dimensions
4  rk = size(LRcsd,2);                        % determine number of factors
5  muREST = mean(DataREST);                   % compute grand mean for REST data
6  sigmaREST = std(DataREST);                 % compute SD for each REST variable
7  LRunit = LRcsd./repmat(sigmaCSD',1,rk);    % rescale LR(csd) to unit
8  LRrest = LRunit.*repmat(sigmaREST',1,rk);  % rescale LR(unit) to LR(rest)
9  Xc = DataREST - repmat(muREST,cases,1);    % remove grand mean
10 FSCFr = LRrest * inv(LRrest' * LRrest);    % compute rotated FS coefficients
11 FSCFr = FSCFr .* ...                       % rescale rotated FS coefficients by
12    repmat(sigmaREST',1,rk);                % ... the corresponding SDs
13 FSrR = zeros(cases,rk);                    % claim memory to speed computations
14 for n = 1:cases                            % for each case ...
15   for m = 1:rk                             % ... compute rotated factor scores from
16      FSrR(n,m) = sum( (Xc(n,:) ./ ...      % ... the normalized raw data and
17        sigmaREST) .* FSCFr(:,m)' );        % ... the corresponding rescaled
18 end; end                                   % ... factor score coefficients
```

Figure A1. Modified Matlab code of function *erpPCA.m* (Kayser and Tenke, 2003).

The critical steps for the back-projection of CSD-PCA factor loadings are summarized in Figure A1. Let *DataREST* be the cases-by-variables surface potential data matrix (i.e., all ERPs)[1] and *LRcsd* the set of 102 Varimax-rotated factor loadings (i.e., variables-by-factors) obtained from the temporal PCA of the CSD-transformed ERP waveforms. Using the known standard deviations of the CSD data (*sigmaCSD*), the CSD factor (covariance) loadings are rescaled to unit (correlation) loadings (*LRunit*, l. 7), and the unit loadings are then rescaled to ERP loadings (*LRrest*, l. 8) using the ERP standard

[1] All ERPs were rereferenced to an estimate of the "infinite" EEG reference using the reference electrode standardization technique (REST; Yao, 2001). However, the particular surface potential reference choice (e.g., REST, linked mastoids, nose, etc.) is irrelevant for the purpose of back-projecting CSD-PCA factors.

deviations (*sigmaREST*). The corresponding factor score coefficient matrix (*FSCFr*) is directly derived from the loadings matrix via matrix multiplication of *LRrest* with the inverse of the transpose of *LRrest* multiplied by *LRrest* (l. 10), and then rescaling *FSCFr* by the ERP standard deviations (*sigmaREST*, l. 11-12). Finally, the factor scores (i.e., for each case and each factor) are derived from the ERP data (after removal of the grand mean, l. 9) and the factor score coefficients (l. 14-18).

This straightforward computation allows recreating the original data, as documented in Figure A2. In fact, any difference between the original and PCA-reconstructed individual 43,776 ERPs (152 subjects x 72 sites x 4 conditions) was minuscule (for all comparisons, $0.99993 \leq r \leq 1.0$) and likely due to computational data precision. Accordingly, the back-projected CSD-tPCA factors can be considered as equivalent estimates of surface potentials, which as such can be subjected to source analysis (e.g., standardized low-resolution brain electromagnetic tomography [sLORETA]; Pascual-Marqui, 2002) without violating the requirement of submitting surface potential topographies (in microvolt [µV]) to the source localization algorithm. This can be in form of virtual ERPs (e.g., Figure A2D) or via a factor score topography that adequately represents a voltage (i.e., ERP) topography.

Appendix References

Kayser J, Tenke CE (2003). Optimizing PCA methodology for ERP component identification and measurement: theoretical rationale and empirical evaluation. *Clin. Neurophysiol.* 114(12):2307-2325.

Kayser J, Tenke CE, Abraham KS, Alschuler DM, Alvarenga JE, Skipper J, et al (2016). Neuronal generator patterns at scalp elicited by lateralized aversive pictures reveal consecutive stages of motivated attention. *NeuroImage* 142:337-350.

Pascual-Marqui RD (2002). Standardized low-resolution brain electromagnetic tomography (sLORETA): technical details. *Methods Find. Exp. Clin. Pharm.* 24:5-12.

Yao D (2001). A method to standardize a reference of scalp EEG recordings to a point at infinity. *Physiol. Meas.* 22(4):693-711.

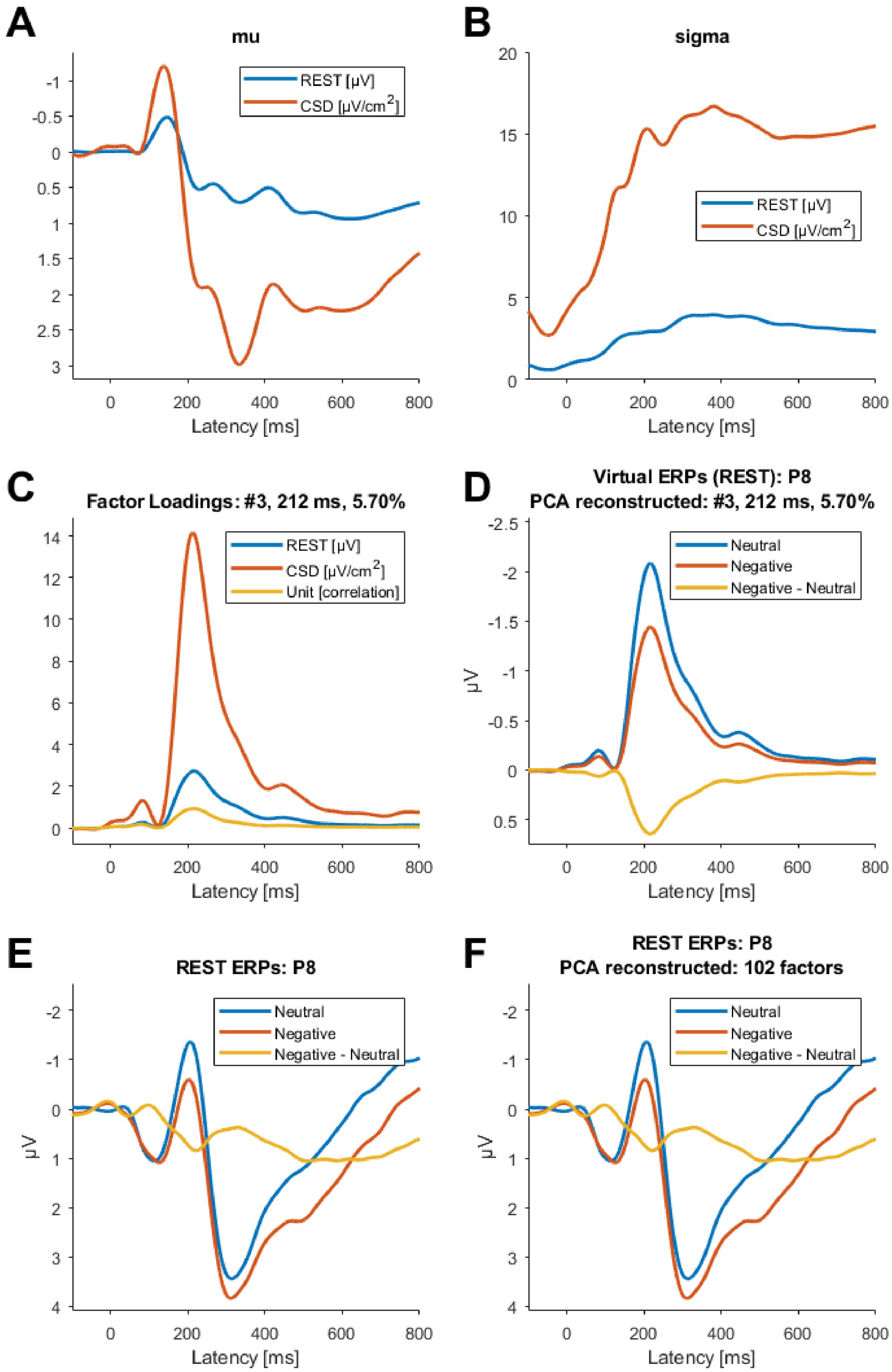

Figure A2. Back-projection of CSD-PCA factor loadings into surface potential (ERP) data space. **A.** Grand means (mu) of ERP [µV] (using the reference electrode standardization technique [REST]; Yao, 2001) and corresponding CSD [µV/cm^2] waveforms (N = 152; see Kayser et al., 2016) computed across all 72 recording sites and 4 conditions (emotional content [2] x visual field [2]). **B.** Standard deviations (sigma) of grand means. **C.** CSD-tPCA factor loadings of factor 3 (*N2 sink*, peak latency 212 ms, 5.7% explained variance) were scaled to unit (correlation) loadings (i.e., by dividing each loading by the corresponding sigma) and then rescaled to ERP (REST) data space (i.e., by multiplying unit loadings with the REST standard deviations). **D.** Virtual ERPs for factor 3 at site P8 (for neutral and negative stimuli and for the respective difference), computed from the REST factor loadings and factor scores, which are calculated from the corresponding factor score coefficient matrix (see text). **E.** Grand mean ERPs of the original REST waveforms. **F.** Grand mean ERPs of the PCA-reconstructed REST waveforms using all 102 factors and reinserting the grand mean (mu), which is removed when employing the covariance matrix for eigenvalue decomposition. The reconstructed ERPs are identical to the original ERPs (all r = 1.0).
